# Evidence-based guidelines in the evaluation of work disability: an international survey and a comparison of quality of development

**DOI:** 10.1186/1471-2458-9-349

**Published:** 2009-09-18

**Authors:** Wout EL de Boer, David J Bruinvels, Arie M Rijkenberg, Peter Donceel, Johannes R Anema

**Affiliations:** 1 Department of Quality of Life, TNO, Hoofddorp, the Netherlands; 2 Department of Public and Occupational Health and EMGO-Institute, Amsterdam, the Netherlands; 3 Department of Quality of Life, TNO, Hoofddorp, the Netherlands; 4 Occup. Med. Service, OCMW-Antwerpen, Antwerp, Belgium; 5 Section of Occupational, Environmental and Insurance Medicine, Department of Public Health, Katholieke Universiteit Leuven, Leuven, Belgium

## Abstract

**Background:**

In social insurance, the evaluation of work disability is becoming stricter as priority is given to the resumption of work, which calls for a guarantee of quality for these evaluations. Evidence-based guidelines have become a major instrument in the quality control of health care, and the quality of these guidelines' development can be assessed using the AGREE instrument. In social insurance medicine, such guidelines are relatively new. We were interested to know what guidelines have been developed to support the medical evaluation of work disability and the quality of these guidelines.

**Methods:**

Five European countries that were reported to use guidelines were approached, using a recent inventory of evaluations of work disability in Europe. We focused on guidelines that are disease-oriented and formally prescribed in social insurance medicine. Using the AGREE instrument, these guidelines were appraised by two researchers. We asked two experts involved in guideline development to indicate if they agreed with our results and to provide explanations for insufficient scores.

**Results:**

We found six German and sixteen Dutch sets of disease-oriented guidelines in official use. The AGREE instrument was applicable, requiring minor adaptations. The appraisers reached consensus on all items. Each guideline scored well on 'scope and purpose' and 'clarity and presentation'. The guidelines scored moderately on 'stakeholder involvement' in the Netherlands, but insufficiently in Germany, due mainly to the limited involvement of patients' representatives in this country. All guidelines had low scores on 'rigour of development', which was due partly to a lack of documentation and of existing evidence. 'Editorial independence' and 'applicability' had low scores in both countries as a result of how the production was organised.

**Conclusion:**

Disease-oriented guidelines in social insurance medicine for the evaluation of work disability are a recent phenomenon, so far restricted to Germany and the Netherlands. The AGREE instrument is suitably applicable to assess the quality of guideline development in social insurance medicine, but some of the scoring rules need to be adapted to the context of social insurance. Existing guidelines do not meet the AGREE criteria to a sufficient level. The way patients' representatives can be involved needs further discussion. The guidelines would profit from more specific recommendations and, for providing evidence, more research is needed on the functional capacity of people with disabilities.

## Background

In the western world, work disability is a problem at the individual, company, and societal levels. Western countries spend about 1.2% of GDP on work disability benefits or 2% if sickness benefits are included, which, for most countries, is an increase over the past 15 years. The probability of returning to work after being granted a long-term disability benefit is below 2% annually on average. Work disability is the end of their working life for the vast majority of recipients [1]. To reduce work disability, many countries have restricted access to disability benefits in social insurance and they have developed programmes to promote return to work [2-4]. In the Netherlands, eligibility criteria have become stricter with the implementation of a new law on long-term work disability. In the United Kingdom, a renewal of the personal capacity assessment for long-term disability benefit was recently implemented [5] and comparable changes are occurring in other countries [2-4]. These policy changes are meant to result in more people being active in work and fewer people receiving disability benefits. In disability benefit systems, social insurance physicians (SIPs) evaluate claims for entitlement to long-term disability benefits [6]. These work disability evaluations are traditionally based mainly on legislation, administrative rules, and doctors' expertise.

When resources are tight, it becomes even more important to determine in a valid and scientifically sound way who is and is not entitled to disability benefit. Internationally, the medical evaluations of work disability turn out to be relatively comparable while being part of social insurance systems that vary strongly [6-8]. The quality of these evaluations is not easy to establish, as no gold standard exists for their validity [9,10]. The mechanism used most often to ensure quality is to organise the process of evaluation in such a way that an optimal result can be expected. A common practice in 14 countries, in Europe and the Russian Federation, is to use qualified doctors, the SIPs, and to have medical reports verified by staff doctors [6]. Although instruments used to support medical decision making are not validated for this purpose [6,9,11], this does not necessarily mean that they are unsuitable.

One way of ensuring the quality of medical work is to use evidence-based guidelines [12], which is common in clinical practice [13]. In clinical practice, guidelines, which the clinician can use with his clinical experience and the patient's preferences, are intended to support the physician by providing recommendations for diagnosis, treatment, and prognosis. [14]. Evidence-based clinical practice means using the best evidence available, in consultation with the patient, to decide on the option that suits that patient best [15]. Guidelines, however, are not restricted to clinical practice: some are being introduced on a wider scale in occupational medicine [16,17] and serve, among other functions, to support the coaching of employees with work-related health problems [18,19]. In occupational medicine, guidelines are intended to provide an occupational physician with recommendations for diagnosis and prognosis of the work-related problem and for the selection of effective interventions [17]. These guidelines can be used in addition to the experience of the occupational health professional and the preferences of the employee and employer. However, guidelines for evaluation in social insurance medicine are a rather new phenomenon.

Having guidelines for medical work does not necessarily mean that the quality of the work is supported. Guidelines need to be adequate for the process they are to support and they need to be used in practice. The Appraisal of Guidelines Research & Evaluation (AGREE) collaboration developed the AGREE instrument to assess the quality of clinical practice guidelines [20] and to establish the quality of the development of guidelines with regard to scientific principles. The AGREE instrument is composed of twenty-three items covering six domains of quality of guideline development: 'scope and purpose', 'involvement of stakeholders', 'rigour of development', 'clarity and presentation of recommendations', 'applicability', and 'editorial independence'. The AGREE instrument has been tested in clinical guidelines and was found to have a good reliability [21]. Thus far, there are no universally accepted cut-off points to identify high-quality guidelines [22]. A high-quality guideline can be expected to contribute to high-quality recommendations but does not warrant them as the evidence used is in general limited and controversial [23,24]. The AGREE instrument is widely used to evaluate clinical guidelines [25,26], as well as those found in occupational medicine [16,27,28], but so far has not been used in social insurance medicine. Social insurance medicine may simply be lagging behind, but the AGREE instrument may not be being used in social insurance medicine because of the rather different medical work involved in social insurance.

Medical practice in social insurance evaluations is different from clinical medical practice in several ways [29,30]. In clinical practice, the consultation is a private initiative of a patient who seeks help that is often restricted by policies of health insurance, whereas in social insurance medicine the consultation is an evaluation that is determined by the legal context and the constraints that the implementing body, the Institution of Social Insurance (ISI), puts on it. In clinical practice, the focus is on disease and finding a cure, whereas in social insurance medicine the focus is on capacity for, and a return to, work. In clinical practice, a patient's request for treatment is taken for granted; in social insurance medicine, the claim to be exempt from work and for a benefit to be paid is scrutinised and evaluated. The position of the claimant in a social insurance context is therefore different from the position of the patient in a clinical care context, differences that have been found to influence the practice of the evaluations [31]. Furthermore, the position of social insurance physicians is different from doctors in clinical medicine as the SIPs have an advisory function towards the ISI they work for and not primarily for the claimant [6]. This position may give rise to tensions between administrative procedures for handling big numbers of claimants and the doctors' need to deliver tailor-made evaluations [32,33].

It is difficult to diagnose the functional consequences of diseases in general and even more so for non-specific diseases such as lower back pain, chronic fatigue, and stress-related disorders. The association between a medical diagnosis and the functional limitations that may lead to work disability is weak and influenced by environmental and personal characteristics, as described in the International Classification of Functioning and Health (ICF) model [34]. From a legal standpoint, evaluations of work disability become more difficult due to stricter eligibility criteria with respect to objectivity, diagnosis, and prognosis of the disability. Sound support from evidence-based guidelines would, therefore, be welcome. The European Union of Medicine in Assurance and Social Security (EUMASS), a network of insurance medicine associations in seventeen European countries, recently published a comparison of work disability evaluation practices and the instruments in use, including guidelines [8]. This comparison was produced by several questionnaire rounds among central medical staff of participating countries. Two central questions in that study were

1. What is evaluated in your countries' work disability evaluation?

2. What instruments are used for these evaluations?

We were interested to determine what guidelines exist in different countries and their quality by focusing on the following research questions:

1. What *disease-oriented *guidelines have been developed to support the medical evaluation of work disability?

2. What is the quality of these guidelines in social insurance medicine?

## Methods

### 1. Identification of disease-oriented guidelines to evaluate work disability

We used the EUMASS table to determine the countries in which guidelines were reported to be in use. The Netherlands, the Czech Republic, Germany, the United Kingdom, and Switzerland were visited based on their reported use of the guidelines; no other countries had reported using guidelines for medical evaluations. The status of guidelines was assessed during the visits by determining if they were officially prescribed. Copies of the guidelines with explanation were collected. For this article we focused on the guidelines for evaluating work disability by SIPs that were prescribed by law or as an instruction by the ISI. We distinguished between disease-oriented guidelines (describing aspects of evaluations for certain pathologies) and process-oriented guidelines (describing aspects of evaluations, regardless of pathology), a distinction that is evident from the relative guideline's title. We selected disease-oriented guidelines. To compare guidelines, we selected those that addressed the same diseases.

### 2. Quality appraisal of guidelines

The selected guidelines were scored using the AGREE instrument, which uses 4-point scales for each item: scope and purpose (3 items), stakeholder involvement (4 items), rigour of development (7 items), clarity and presentation of the recommendations (4 items), applicability of the guideline (3 items), and editorial independence (2 items). To correct for the different number of items in each domain, The AGREE instruments suggests calculating domain scores by relating the obtained scores (OS) to the maximum possible score (MaPS) and the minimum possible score (MiPS) using the formula



As a test, one (Dutch) guideline (burnout) was scored by two researchers (WdB and DB) using the AGREE instrument and its user guide to establish if additional rules for scoring would be required. The test showed the need for additional scoring rules. We specified the clinical question and the target population and we adapted user guide item 11 (health benefits, side effects and risks) and 16 (options for management of the condition) [see Additional File 1]. The selected guidelines were then scored independently by two researchers (WdB and DB). The initial agreement between the researchers was determined using Kappa. Any differences were discussed, but if a difference remained, a decisive third researcher (JRA) would score as well, using the scores and arguments of the first two. We analysed the initial correlation between the two scoring researchers. As this use of the AGREE instrument is new in social insurance medicine, we asked one expert in each country who had participated in developing several guidelines for a reaction to our results: "Are these correct in your view and what is your explanation for any insufficient scores?"

### Ethics committee

This study was not submitted for ethical approval. The study included physicians who were not asked to perform specific professional actions for this study, but only to complete a questionnaire. All studied documents are in the public domain.

## Results

### 1. Identification of disease-oriented guidelines to evaluate work disability

In Germany seven guidelines for SIPs turned out to be officially in use. In the Netherlands twenty-four were found and one in Switzerland. These guidelines are partly process-oriented and partly disease-oriented. Process guidelines were used in Germany (1), the Netherlands (8), and Switzerland (1). The German and Swiss guidelines each contain many recommendations that in the Netherlands are distributed over eight smaller guidelines. The recommendations refer, for example, to the relevance of the diagnosis for the evaluation and to the boundaries of the concept of disease. Another topic of these guidelines is the claimant's obligation to attempt to recover and find gainful employment. Yet another aspect is the relevance of distinguishing between the opinions of the claimant and the SIP. These recommendations represent the consensus of legal and medical experts on the principles of evaluation, but not on scientific evidence. These process-oriented guidelines were excluded.

Disease-oriented guidelines were in use in Germany (6) and the Netherlands (16), shown in Table 1. In the Czech Republic, a Barema-type of guideline is in official use, but this was excluded from this study as it evaluates impairments, not work disability.

**Table 1:** Diagnosis-oriented guidelines for SIPs to country, publisher, and year of publication/revision, nr of pages (exc summary and addenda) and nr of references.

**Guideline (country and publisher)**	**Year**	**Pages**	**References**
Aspecific Lumbar Disorder (NL, Health Council)	2005/2008	20	15

Myocardial Infarction (NL, Health Council)	2005/2008	22	45

Anxiety Disorders (NL, Health Council)	2007	30	27

Stroke (NL, Health Council)	2007	30	30

Breast Cancer (NL, Health Council)	2007	24	35

Chronic Fatigue Syndrome (NL, Health Council)	2007	26	22

Herniating Intervertebral Disc (NL, Health Council)	2007	20	14

Burnout (NL, Health Council)	2007	28	29

Depressive Disorder (NL, Health Council)	2007	32	29

Whiplash Associated Disorders (NL, Health Council)	2008	26	24

Arthritis Hip and Knee (NL, NVVG)	2007	28	56

Rheumatoid Arthritis (NL, NVVG)	2007	34	57

Chronic Obstructive Lung Disease (NL, NVVG)	2007	46	54

Chronic Heart Failure (NL, NVVG)	2007	30	41

Schizophrenia and associated psychoses (NVVG)	2007	50	135

Chronic Shoulder Disorders (NL, NVVG)	2007	21	37

			

Mental disorders (DE, DRV)	2001/2006	53	59

Herniating Intervertebral Disc (DE, DRV)	2002/2003/2005	26	28

Chronic Inflammatory Bowel Disease (DE, DRV)	2005	26	60

Coronary Heart Disease (DE, DRV)	2001/2005	20	54

Chronic Obstructive Lung Disease (DE, DRV)	2003/2005	34	47

Breast Cancer (DE, DRV)	2006	22	42

The Dutch guidelines, all implemented by law, were first developed by the Health Council of the Netherlands and later by the scientific association of SIPs (NVVG). The German guidelines were developed and prescribed by the German Institution of Social Insurance (DRV). The German guidelines were developed earlier than the Dutch and most have been updated since their inception.

### 2a. The appraisal of quality with the AGREE instrument of selected guidelines

Of the guidelines, four diseases were common to both countries: breast cancer, chronic obstructive lung disease, lumbar intervertebral disc herniation, and myocardial infarction.

The initial agreement between researchers was high for the Dutch guidelines (Kappa range 0.814-0.939), but low for the German counterparts (Kappa range 0.449-0.624). After discussing the different opinions of the researchers, agreement was reached on all items and scoring by the third researcher was unnecessary. The results are presented in Table 2.

**Table 2:** AGREE scores of selected guidelines to domain

	**Breast Cancer**		**Chronic Obstructive Lung Disease**		**Lumbar Intervertebral Disc Herniation**		**Myocardial Infarction**		**Total**	
	**Dutch**	**German**	**Dutch**	**German**	**Dutch**	**German**	**Dutch**	**German**	**Dutch**	**German**

*Scope and purpose of the guideline*	100	100	100	100	100	100	100	100	100	100

*Stakeholder involvement*	58	33	50	33	50	33	50	33	52	33

*Rigour of development*	10	19	19	24	19	19	14	29	16	23

*Clarity and presentation*	75	67	75	67	50	75	50	75	63	71

*Applicability*	11	0	11	33	0	0	0	0	6	8

*Editorial independence*	50	0	50	0	50	0	50	0	50	0

*The scope and purpose of the guideline *were well described in all eight guidelines; the score in both countries was **100%**. All guidelines were designed to support the medical evaluation of work disability by indicating what functional incapacities were to be expected in cases with a specific diagnosis.

*Stakeholder involvement *was **52% **for the Dutch and **33**% for the German guidelines. Potential users were well defined (social insurance physicians), but the involvement of professional groups was found to be incomplete in seven of the eight guidelines. The patients' views were not sought in the German guidelines and only at the final stage in the Dutch. No guidelines were piloted among end-users before their publication.

*Rigour of development *was **16% **with the Dutch and **23% **with the German guidelines. How evidence was gathered and the scientific grounding of recommendations were not explicit in any guideline.

*Clarity and presentation *of the guidelines was **63% **for the Dutch guidelines and **71% **for the German. Although the recommendations were unambiguous and easily identifiable in almost all cases, they were not overly specific. Different options for assessing the condition of the guidelines were often mentioned, and the German guidelines provided tools for the evaluations.

*Applicability *scored **6% **in the Netherlands and **8% **in Germany. Practical barriers and costs were not addressed in any guideline. The German guidelines contained indications of when to update them.

*Editorial independence *was limited in both countries. The Dutch guidelines reached **50% **on average as they were developed independently of the funding body, but with only a general procedure about conflicting interests. The German guidelines (**0%**) were developed entirely within the ISI and conflicting interests were not addressed.

### 2b Feedback on the AGREE scores by experts involved in developing several guidelines

The Dutch expert was involved in developing 11 of 16 then-published guidelines in the Netherlands and 3 of the 4 protocols that we scored on the AGREE instrument. He agreed to all our scoring after we discussed our scoring rules with him. He attributed low scores to the newness of creating guidelines for social insurance medicine in the Netherlands and that the short time allotted to create them was a factor. Stakeholder involvement was also reduced because patients' involvement was controversial in the beginning as there was concern about patients being biased with regard to the recommendations. The low figure on rigour of development was because the methods of development had not been recorded and because the field had no scientific tradition. The lack of specificity of the recommendations was due mainly to a lack of existing scientific research. Applicability scored low in the Netherlands as the guidelines were developed by the Health Council, for whom this was not a regular activity. The aspects of applicability were considered by the ISI after publication of the guidelines.

The German expert was involved in developing five of six guidelines published at the time in Germany and in all the guidelines we scored on the AGREE instrument. He agreed to nineteen of the twenty-three scores after we discussed our scoring rules with him. Differences were due partly to how the German guidelines were described (experts involved were not identified with their specialisation) and to differences in the interpretation of items 13, 14, and 15. He commented that the development of guidelines was new in Germany and started from a need of the SIPs within the ISI, which explained the limited involvement of stakeholders. The involvement of patients' representatives was considered unhelpful because of expected bias. Testing among users was done implicitly as the guidelines were developed at the institution where the SIPs work. The selection of evidence and formulation of recommendations were carried out according to what the German experts considered the most important. No need had existed to document any more than they did for internal use, which accounted for the low score on the rigour of the guidelines' development. This internal development also accounted for the low score on applicability; this was included implicitly within the development process of internal guidelines. Editorial independence was not considered important, as the interests of the SIPs and the ISI were not supposed to conflict.

## Discussion

In this study we looked for the existence of evidence-based guidelines for the medical evaluation of long-term work disability and the quality of development of these guidelines.

### Main findings

Using the EUMASS comparison, we found guidelines for the medical evaluation of work disability, both disease- and process-oriented, in official use in four of seventeen European countries. In two of these countries we found twenty-two disease-oriented guidelines in official use in these evaluations. The AGREE instrument was applicable for scoring the selected Dutch and German guidelines, although minor adaptations to the AGREE instrument were necessary. Scoring German guidelines gave a smaller initial agreement than the Dutch, due to language problems and understanding of the German social insurance; however, the consensus procedure compensated for these issues. The guidelines scored well on 'scope and purpose' and 'clarity and presentation', and moderately on 'stakeholder involvement' in the Netherlands, but low in Germany; all guidelines scored low on 'rigour of development'. 'Editorial independence' and 'applicability' were low as a result of how production was organised.

### Strengths and weaknesses

To our knowledge, this is the first study to identify and qualify medical guidelines in social insurance medicine at an international level. As we were looking for official guidelines, we do not believe that we missed any in the countries we included; however, focusing on official guidelines may have resulted in finding fewer guidelines than are in practical use. For example, in Germany and Switzerland, guidelines are published by specialists in scientific journals. These are not in official use, but they may support physicians in their evaluations.

We used the AGREE instrument to determine the quality of the guidelines, which recommends using four appraisers for a good reliability [20]. Using a pilot procedure and two researchers for scoring, we obtained good agreement, which was supported by the opinion of the two experts who were involved in developing the guidelines. All items of the AGREE instrument proved to be relevant for testing the guidelines. We did not encounter important aspects that were not addressed by the AGREE instrument; further validation is needed however. Our adaptations are partly specifications of the scope of the AGREE instrument to the context of social insurance medicine, but are unlikely to influence the integrity of the AGREE instrument. Our adaptations of items 11 and 16 are less clear-cut translations that need to be tested.

### Other studies

Our study corresponds with other research; the distinction between legal and medical guidelines fits with the results of Boer et al. [35] about the medical and legal aspects of a doctor's reasoning. The reliability of the AGREE instrument outside the clinical domain [16,27,28] was partly confirmed in our study, after minor alterations were made. Finding that guidelines do not fully meet the AGREE criteria is not uncommon [22,36-38], partly due to the lack of a precise account of the development process and partly because of a lack of scientific evidence; both are not uncommon problems in drafting guidelines [22,39,40]. The relative lack of scientific research on the work participation of people with chronic diseases is also well documented [40-43].

### Impact

We found disease-oriented guidelines in only two participating countries, and there they are recent. Work disability is being evaluated on similar aspects in many countries, despite large differences in organisation of social insurance [6]; thus, we expect the development of guidelines to be likely elsewhere. Our results may be helpful in facilitating this.

Our comparison of development quality is based on four Dutch and four German guidelines, on four different pathologies. The German and Dutch social insurance systems differ in many aspects, but both require a medical statement about functional capacity in cases of claims for work disability benefit. From this perspective the guidelines are comparable in and between countries. As the guidelines in these countries have been created in a similar fashion, we expect our results to be relevant to future disease-oriented guideline development in these countries.

We used the AGREE instrument as a tool for evaluating the quality of guideline development in social insurance medicine, a procedure that, to our knowledge, is new. It is unclear if using the AGREE instrument in a different domain is without problems; however, neither we, nor the experts we consulted, noticed any clear incongruence. The AGREE instrument is now being utilised in both Germany and the Netherlands.

With the AGREE instrument the quality of the development of guidelines can be scored, which is not the same as the quality of the recommendations. It is possible that the guidelines contain adequate recommendations that have been developed in a suboptimal way or whose development has been accounted for in a suboptimal way. Good practice, however, is best supported by guidelines that have been developed in a proven, optimal way. Several aspects need further consideration. The involvement of patients' representatives is now accepted in the Netherlands, after much discussion about the nature of their input; in Germany, however, this is not the case. This difference illustrates the ambiguity of the claimant's position in social insurance medicine: he is both passive object of the evaluation and participating subject in work disability. AGREE criteria are clear, however: participation of patients' representatives is mandatory. The development of the guidelines in the Netherlands has now been placed under the authority of the scientific association of SIPs, as this is viewed as the best way to retain independence from both the funding and implementing bodies. In Germany, financing, developing, and implementing within the ISI is considered effective, which illustrates the ambiguity of the profession of social insurance medicine as a discipline that needs to stress its independence and quality and a group of doctors working for administrative organisms with more interests than medical quality [29,33]. AGREE criteria are clear on this aspect, too: a good guideline needs to be developed independently.

The inclusion of disease-oriented research into the practice of disability evaluation will help coordinate clinical, occupational, and social insurance medicine, in using the same concepts and findings, although in different spheres. The lack of scientific evidence may be compensated for, in part, by research on the aspects that influence disability with chronic conditions in general [41,43]. Parallel to this, research needs to be commenced to establish if the guidelines actually contribute to quality improvement. Finally, the production of these guidelines will help formulate the questions that need to be addressed in future research to ground social insurance evaluations.

We expect that the diffusion of our results may aid further development of guidelines in social insurance medicine and, notably, help these become increasingly more evidence-based, which would assist in establishing a new and important mechanism for quality control in social insurance medicine. Paraphrasing Lohr [15], evidence-based evaluation practice in social insurance medicine would mean using the best evidence available and the best procedure possible to decide on the option that suits that claimant best.

## Conclusion

Evidence-based guidelines form an important instrument for enhancing the quality of medical practice. Guidelines can provide a framework on which a clinician can ground diagnosis, therapy, and prognosis. Guidelines in social insurance medicine for the evaluation of work disability are a recent phenomenon, so far restricted to Germany and the Netherlands. We expect that disease-oriented guidelines can be useful in other countries as well, and can help the SIP ground his evaluation of capacity for work. For the practice of evaluating work disability, this would mean an important instrument to control quality. The AGREE instrument is suitably applicable for assessing the quality of guideline development in social insurance; nevertheless, some of the scoring rules need to be adapted to the context of social insurance. Existing guidelines do not meet AGREE criteria sufficiently. Notably, how patients' representatives can be involved and the editorial independence of the guideline developers need further discussion. The guidelines would profit from more specific recommendations and, for this, more research is needed on the functional capacity of people with disabilities. To date, research has focused primarily on the recovery from complaints, while mainly ignoring the resumption of work. The latter depends on much more than a health condition, but still, the challenge of health care should not only be to give relief for pain and suffering, but also to allow participation in society and to legitimise a disability benefit if needed for medical reasons.

## Competing interests

The authors declare that they have no competing interests.

## Authors' contributions

WB designed the study, carried out the field work, and prepared the manuscript. DB participated in the scoring and drafting of the article. AR participated in the field work. PD supervised the field work and participated in drafting the article. HA supervised and participated in the drafting of the article. All authors read and approved the final manuscript.

## Pre-publication history

The pre-publication history for this paper can be accessed here:



## Supplementary Material

Additional file 1 **AGREE from clinic to social insurance**. this document describes the way in which AGREE criteria were used in the study of guidelines in social insurance. Click here for file
