# Supplementary material for: Evidence-based guidelines in the evaluation of work disability: an international survey and a comparison of quality of development
Source: BMC Public Health. 2009 Sep 18;9:349. doi: 10.1186/1471-2458-9-349 (PMC2754463; doi:10.1186/1471-2458-9-349)
Supplement: Additional file 1 — AGREE from clinic to social insurance. this document describes the way in which AGREE criteria were used in the study of guidelines in social insurance. [file 1471-2458-9-349-S1.DOC]

**Additional file 1**

AGREE from clinic to social insurance:

Most AGREE items were found to be directly applicable to the guidelines in social insurance medicine. We specified the AGREE items 2 and 3 and adapted items 11 and 16 from a clinical context to a context of evaluation in social insurance medicine.

Scope and purpose

1 The overall objective(s) of the guideline is (are) specifically decribed.

2 The clinical question(s) covered by the guideline is (are) specifically described.

*We specified the clinical question as: what functional incapacities are to be expected with diagnosis X?*

3 The patients to whom the guideline is meant to apply are specifically described.

*We specified the target population as: the claimants to whom the guidelines should be applied.*

Stakeholder involvement

4 The guideline development group includes individuals from all the relevant professional groups.

5 The patients’ view and preferences have been sought.

6 The target users of the guideline are clearly defined.

7 The guideline has been piloted among target users

Rigour of development

8 Systematic methods were used to search for evidence

9 The criteria for selecting the evidence are clearly described.

10 The methods used for formulating the recommendation are clearly described.

11 The health benefits, side effects and risks have been considered in formulating the recommendations.

*We considered the risks of following or not following of the guideline as criterion.*

12 There is an explicit link between the recommendations and the supporting evidence.

13 The guideline has been externally reviewed by experts prior to its publication.

14 A procedure for updating the guideline is provided.

Clarity and presentation

15 The recommendations are specific and unambiguous

16 The different options for management of the condition are clearly presented.

*We decided to score if the guideline indicates differences in recommendations in different situations.*

17 Key recommendations are easily identifiable

18: The guideline is supported with tools for application.

Applicability

19 The potential barriers in applying the recommendations have been discussed.

20 The potential cost implications of applying the recommendations have been considered

21 The guideline presents key review criteria for monitoring and/ or audit purposes.

Editorial independence

22 The guideline is editorially independent from the funding body.

23 Conflicts of interest of guideline development members have been recorded
